# Differential expression of anti-angiogenic factors and guidance genes in the developing macula

**Published:** 2009-01-12

**Authors:** Peter Kozulin, Riccardo Natoli, Keely M. Bumsted O’Brien, Michele C. Madigan, Jan M. Provis

**Affiliations:** 1 ARC Centre of Excellence in Vision Science and School of Biology, The Australian National University, Canberra, Australia; 2 School of Optometry and Vision Science, The University of New South Wales, Kensington, and Save Sight Institute, The University of Sydney, Sydney, Australia; 3 Australian National University Medical School, The Australian National University, Canberra, Australia

## Abstract

**Purpose:**

The primate retina contains a specialized, cone-rich macula, which mediates high acuity and color vision. The spatial resolution provided by the neural retina at the macula is optimized by stereotyped retinal blood vessel and ganglion cell axon patterning, which radiate away from the macula and reduce shadowing of macular photoreceptors. However, the genes that mediate these specializations, and the reasons for the vulnerability of the macula to degenerative disease, remain obscure. The aim of this study was to identify novel genes that may influence retinal vascular patterning and definition of the foveal avascular area.

**Methods:**

We used RNA from human fetal retinas at 19–20 weeks of gestation (WG; n=4) to measure differential gene expression in the macula, a region nasal to disc (nasal) and in the surrounding retina (surround) by hybridization to 12 GeneChip^®^ microarrays (HG-U133 Plus 2.0). The raw data was subjected to quality control assessment and preprocessing, using GC-RMA. We then used ANOVA analysis (Partek^®^ Genomic Suite™ 6.3) and clustering (DAVID website) to identify the most highly represented genes clustered according to “biological process.” The neural retina is fully differentiated at the macula at 19–20 WG, while neuronal progenitor cells are present throughout the rest of the retina. We therefore excluded genes associated with the cell cycle, and markers of differentiated neurons, from further analyses. Significantly regulated genes (p<0.01) were then identified in a second round of clustering according to molecular/reaction (KEGG) pathway. Genes of interest were verified by quantitative PCR (QRT–PCR), and 2 genes were localized by in situ hybridization.

**Results:**

We generated two lists of differentially regulated genes: “macula versus surround” and “macula versus nasal.” KEGG pathway clustering of the filtered gene lists identified 25 axon guidance-related genes that are differentially regulated in the macula. Furthermore, we found significant upregulation of three anti-angiogenic factors in the macula: pigment epithelium derived factor (*PEDF*), natriuretic peptide precurusor B (*NPPB*), and collagen type IVα2. Differential expression of several members of the ephrin and semaphorin axon guidance gene families, *PEDF*, and *NPPB* was verified by QRT–PCR. Localization of *PEDF* and *Eph-A6* mRNAs in sections of macaque retina shows expression of both genes concentrates in the ganglion cell layer (GCL) at the developing fovea, consistent with an involvement in definition of the foveal avascular area.

**Conclusions:**

Because the axons of macular ganglion cells exit the retina from around 8 WG, we suggest that the axon guidance genes highly expressed at the macula at 19–20 WG are also involved in vascular patterning, along with *PEDF* and *NPPB*. Localization of both *PEDF* and *Eph-A6* mRNAs to the GCL of the developing fovea supports this idea. It is possible that specialization of the macular vessels, including definition of the foveal avascular area, is mediated by processes that piggyback on axon guidance mechanisms in effect earlier in development. These findings may be useful to understand the vulnerability of the macula to degeneration and to develop new therapeutic strategies to inhibit neovascularization.

## Introduction

**Figure 1:**
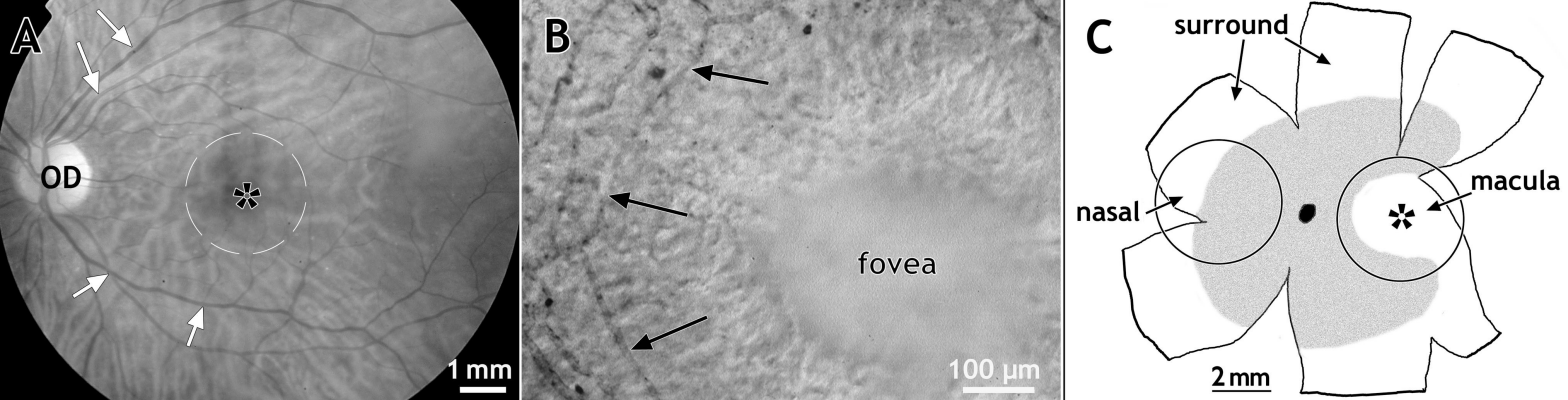
Adult and fetal human retinas. **A:** Image showing the fundus of an adult human eye with the optic disc (OD), the macula (broken circle) and the location of the fovea centralis (asterisk) indicated. The large retinal vessels extend from the optic disc into the supero-temporal and infero-temporal retina (white arrows), but do not grow directly toward the macula. Only fine caliber vessels are present in the macula. **B:** High power micrograph showing microvessels (black arrows) surrounding the fovea centralis in a retinal flatmount. The fovea is a shallow depression, <1 mm in diameter, in the surface of the retina, located at the center of the macula, and centered on an avascular area 500–700 μm in diameter. Absence of capillaries at the fovea ensures that the highest quality image possible reaches the photoreceptors. **C:** A diagram of a flatmounted human fetal retina at 19 WG showing the vascularized region of the retina at this stage in gray, and the location of the fovea (asterisk). The two circles indicate the approximate size and location of biopsies taken from nasal and temporal retina, and used for RNA extraction for this study. RNA was also extracted from the remainder of the retina (surround).

A unique adaptation of the central retina in monkeys and humans is the distinctive macula, at the center of which is the highly specialized fovea centralis (fovea; Figure 1). Within the macula the density of all neural elements is elevated and there are no large retinal vessels. At the fovea there are no rods, but there is a peak spatial density of cone photoreceptors, and a prevalence of “midget” circuitry that mediates high acuity and color vision [1]. Several retinal diseases specifically affect the macula, including Best's disease, macular telangiectasia, Stargardt's macular dystrophy, and age-related macular degeneration (AMD). It is not known why the macula is the focus of these disorders. AMD is the most common cause of untreatable blindness, resulting from degeneration of macular photoreceptors, with or without neovascularization [2]. While recent gene association studies attribute high risk for AMD to a relatively small number of SNPs [3-6], why the disease specifically affects the macula remains poorly understood. Because of the vulnerability of the macula, and the high impact consequences of macular disease, there has been considerable interest in identifying genes differentially expressed in the macula and foveal region [7-10]. To our knowledge, however, no studies have investigated differential gene expression in the macula during development, that is, during the period when genes that are preferentially associated with determination of macular characteristics are most likely to be expressed.

In all species examined, the fovea is found within an area devoid of retinal vessels - the foveal avascular area (Figure 1) [11]. In humans retinal vessels enter the developing human retina in the 14th week of fetal life (14 weeks of gestation; WG) and grow slowly toward the developing macula, compared with the rest of the retina [12]. In normal development, the foveal region is never vascularized [13], and absence of a foveal avascular region is associated with foveal hypoplasia [14,15]. During fetal life, the macula is the most developmentally advanced region of the neural retina, and the principal angiogenic factor, vascular endothelial growth factor (VEGF), is highly expressed by ganglion cells in the macula [16]. Despite this, blood vessels growing into the macular region proceed slowly [12], have lower rates of proliferation [17], and appear to face a molecular barrier that prevents formation of vessels at the incipient fovea [18,19]. Developmental studies of macaque and marmoset retinas also indicate that the fovea forms only after the foveal avascular area has been defined [18,20].

The photoreceptor layer is normally avascular throughout life, and neovascularization of the outer retina is associated with vision loss, as in exudative AMD. It is thought that a critical balance between the pro-angiogenic factor *VEGF* and the anti-angiogenic pigment epithelial derived factor (*PEDF*) is essential for preserving the avascular environment of the outer retina [21]. Both of these factors are expressed by the retinal pigmented epithelium. However, to date there have been no reports of differential expression of *PEDF* or other anti-angiogenic factors that may be involved in definition of the foveal avascular area, even though data from several gene expression studies of adult retina have been reported [9,10]. Furthermore, because guidance factors commonly define “no-go” zones for axons, and members of the ephrin, netrin, slit, and semaphorin families have roles in both axon and vascular guidance [22], we hypothesized that a gradient of guidance molecules centered on the incipient fovea might also participate in definition of the foveal avascular area. Our aim, therefore, was to determine if genes associated with anti-angiogenic regulation as well as axon and vascular guidance are differentially expressed in the macula during development.

## Methods

### Human retinas

Human retinas (n=18) were obtained at surgery to terminate pregnancy from fetuses between 17 and 20 WG, with informed maternal consent and ethical approval from Human Ethics Committees of The University of Sydney and The Australian National University. Gestational age was determined by ultrasound before surgery and confirmed by postmortem measurements of foot length.

### RNA extraction

For hybridization to microarrays (4 retinas), eyes were removed and stored in separate solution-free containers, on ice, prior to dissection. RNA was extracted from these retinas 90-120 min post mortem. For QRT-PCR, whole eyes were placed in TRIzol Reagent (Invitrogen, Carlsbad, CA) and frozen at -80 °C for subsequent RNA extraction, up to one month post mortem. The anterior segment and vitreous were dissected away, and the eye cup was flattened by making radial cuts through the sclera and retina. A 5 mm trephine was used to take two biopsies of each retina: one from the posterior pole (“macula” sample), and a second from the corresponding region immediately nasal to the optic disc (“nasal” sample). The remaining retina was used as a third sample region (“surround” sample). The biopsied regions are illustrated in Figure 1C. Each of the three retinal biopsies (macula, nasal, and surround) was homogenized then combined with TRIzol^®^ Reagent (Invitrogen, Carlsbad, CA) and chloroform to separate the RNA from other cell components. The RNA was then purified and isolated using RNAqueous^®^-Micro RNA isolation kit (Ambion, Inc., Austin, TX). RNA concentration was determined using a NanoDrop^®^ ND-1000 spectrophotometer (NanoDrop Technologies, Wilmington, DE). The RNA Integrity Number (RIN – determined by the Agilent Bioanalyzer 2100 eukaryotic total RNA Nano assay, Agilent Technologies, Inc., Santa Clara, CA) was used as a measure of the quality of the RNA [23]. In some cases where genomic DNA contamination was a concern, the RNA was DNase treated [24]. This treatment did not affect the RIN.

### Microarray

Three sample regions (macula, nasal, and surround) from each of four retinas, three at 19 WG and one at 20 WG were used for microarray analysis. Double-stranded cDNA was generated from each of the twelve total-RNA samples and transcribed to obtain biotin-labeled cRNA. The target cRNAs were fragmented, combined with hybridization and spike controls, then hybridized to the twelve HG-U133 Plus 2.0 GeneChip^®^ microarrays (Affymetrix, Inc., Santa Clara, CA) for 16 h. Because of the limited availability of suitable donor retinas, six cDNA samples (from two retinas) were hybridized to microarrays on two separate occasions, that is, the hybridizations were performed in two batches. Following hybridization, the arrays were washed and stained with streptavidin phycoerythrin conjugate using an automated GeneChip^®^ Fluidics Station 450, and then scanned with a GeneChip^®^ Scanner 3000 using a 570 nm excitation wavelength laser. Target sample preparation and microarray hybridization and scanning were performed at the Biomolecular Resource Facility, John Curtin School of Medical Research, The Australian National University (Canberra, Australia).

To allow correct alignment of the software analysis grid, a control oligo (B2 oligo) was hybridized and used to indicate the boundaries of the probe area. Other quality control metrics used include average background and noise (Raw Q) of the measured probe cell intensities, ratio of expression of the 3′ probe set to 5′ probe set of the housekeeping control gene glyceraldehyde-3-phosphate dehydrogenase (*GAPDH*), and the percentage of all probe sets on the array detected as present. Poly(A) sense RNA labeling controls and antisense biotinylated cRNA hybridization controls (together with the B2 oligo) were also spiked into the total RNA and biotinylated cRNA cocktails, respectively, at known concentrations before array hybridization (Affymetrix, Inc., GeneChip Expression Analysis technical manual; 2003). The poly(A) RNA controls were generated from four cloned *B. subtilis* genes (*lys, phe, thr*, and *dap*), and the hybridization controls transcribed from three *E. coli* genes (*bioB, bioC*, and *bioD*) and one P1 bacteriophage recombinase gene (*cre*).

The perfect match (PM) and mismatch (MM) probe intensities were used to generate a detection call (present, marginal, or absent) for each probe set, to assess the reliability of detection of a transcript. Detection calls were generated by the microarray suite version 5 (MAS5) algorithm. Microarray quality controls were assessed using GeneChip® Operating Software (Affymetrix).

### Data analysis - preprocessing

Affymetrix CEL files were imported into Partek^®^ Genomics Suite™ 6.3 (Partek Inc., St. Louis, MO) using the default Partek normalization parameters. Probe-level data was pre-processed, including background correction, normalization, and summarization [25], using robust multi-array average (RMA) analysis adjusted for probe sequence and GC content (GC-RMA). GC-RMA adjusts for background noise on each array using only the PM probe intensities; subsequent data normalization was performed across all arrays using quantile normalization [26,27]. The background-adjusted, normalized PM values were then compiled, or summarized, within each probe set, using the median polish technique, to generate a single measure of expression [27]. These expression measures were then log transformed, base 2.

### Data analysis - differential expression analysis

Differential expression analysis was performed using a two-way ANOVA in which the variables were sample region (macula, nasal, and surround) and hybridization batch. No significant batch effect was evident in the data. Data from the four macula arrays were grouped and compared (using a linear contrast) with either the four nasal or surround arrays as baselines. The lists of differentially expressed genes from the macula versus surround and macula versus nasal data were clustered according to functional roles or biological process as specified by the Gene Ontology Consortium [28]. This was achieved using the functional annotation tool on the Database for Annotation, Visualization, and Integrated Discovery (DAVID) website [29] with an Expression Analysis Systematic Explorer (EASE) score threshold (a modified Fisher Exact p-value) of 0.1.

The retina differentiates in a gradient which progresses from the macula to the periphery. At 19–20 WG (the ages of our samples), the majority of cells in the macula have exited the cell cycle and differentiated as neurons, while the majority of cells in non-macular locations are still proliferating [30]. Because of these significant differences in cell function between macular and non-macular locations, we excluded genes that clustered under the general heading of “biological processes” from further differential analysis. The remaining list of genes was then subcategorized according to the molecular/reaction pathways described by the Kyoto Encyclopedia of Genes and Genomes (KEGG) using the DAVID web tool and 0.1 EASE score threshold.

### Quantitative real time polymerase chain reaction

cDNA from human retinas aged between 17 and 20 WG were used for evaluative quantitative real-time polymerase chain reaction (QRT–PCR) analyses. First-strand cDNA synthesis was performed in a 20 μl reaction mixture using 1 μg RNA, 500 ng oligo (dT)_18_ primer, and 200 U SuperScript™ III reverse transcriptase (Invitrogen). Amplification of *Eph-A6* and *ephrin A1–5* was measured using SYBR^®^ GreenER™ (Invitrogen). All other genes were amplified using commercially available TaqMan^®^ probes (Applied Biosystems, Melbourne, Australia). Primers were designed within the coding domain sequence and across an intron using reference sequences (RefSeq) derived from the NCBI Entrez Gene database and the OligoPerfect™ Designer web-based program (Invitrogen). Primer sequences and TaqMan probe IDs are provided in Table 1 and Table 2, respectively. Fluorescence was measured on a FAM 510 nm detection channel using a commercial device (Rotor-Gene RG-3000; Corbett Research, Sydney, Australia) and normalized relative to a glyceraldehyde-3-phosphate dehydrogenase (*GAPDH*) reference gene. TaqMan amplification was performed in duplicate, and SYBR GreenER PCR runs in triplicate, using cDNA from three different specimens (total of 6 and 9 reactions per gene, respectively). Amplification specificity was assessed using either melt curve analysis (SYBR GreenER) or gel electrophoresis (TaqMan). The results were analyzed using the relative quantification method developed by Pfaffl [31].

**Table 1:** QRT–PCR primers

**NCBI RefSeq**	**Gene**	**Forward primer (5′-3′)**	**Reverse primer (5′-3′)**
NM_001080448	*Eph-A6*	TTTTTCTCCCAAGCCATTCA	ATGCCCAGTCCTTCCTTACC
NM_004428	*ephrin-A1*	GGTGACTGTCAGTGGCAAAA	GCACTGTGACCGATGCTATG
NM_001405	*ephrin-A2*	GCCTGCGACTGAAGGTGTA	CGGGCTGCTACACGAGTTAT
NM_004952	*ephrin-A3*	ACTCTCCCCCAGTTCACCAT	GTCCCGCTGATGCTCTTCT
NM_005227	*ephrin-A4*	ACTACATCTCGGTGCCCACT	CTGATGTGCCACTCTCTCCA
NM_001962	*ephrin-A5*	TTCATGATCGTGTTTTCGATG	GCTGGGTATCCTTGGTGTTT
NM_002046	*GAPDH*	TGCACCACCAACTGCTTAGC	GGCATGGACTGTGGTCATGAG

QRT–PCR primers were designed to verify gene expression of *Eph-A6* and its ligands *ephrin-A1* and *-A4*, as well as *ephrin-A2*, -*A3,* and *-A5*. These genes were amplified using a SYBR GreenER QRT–PCR kit (Invitrogen) and amplification was normalized relative to a *GAPDH* reference gene. QRT–PCR reactions were done in triplicate and repeated using cDNA from three different specimens.

**Table 2:** TaqMan probes

**Entrez Gene ID**	**Gene**	**TaqMan probe ID**	**Amplicon length**
Axon guidance genes			
22854	*NTNG1*	Hs01552822_m1	61
8829	*NRP1*	Hs00826129_m1	97
91584	*PLXNA4*	Hs00326001_m1	89
10154	*PLXNC1*	Hs00194968_m1	72
223117	*SEMA3D*	Hs00380877_m1	97
56920	*SEMA3G*	Hs00928864_m1	106
10505	*SEMA4F*	Hs00188642_m1	64
137970	*UNC5D*	Hs00369888_m1	83
Regulators of Angiogenesis			
4879	*NPPB*	Hs00173590_m1	82
5176	*PEDF*	Hs01106937_m1	84
7422	*VEGFA*	Hs00900057_m1	81
Other			
2247	*FGF2*	Hs00266645_m1	82
2264	*FGFR4*	Hs00242558_m1	74
Control			
2597	*GAPDH*	Hs99999905_m1	122

QRT–PCR amplification of genes not listed in Table 1 was performed using TaqMan probes (Applied Biosystems) and normalized relative to a *GAPDH* reference gene. *GAPDH* gene expression was also measured using a TaqMan probe.

### Macaque retinas and in situ hybridization

For localization studies we used histological sections of retinas from eight macaques of known gestational ages: fetal day (Fd) 80, Fd 115, and Fd 150. The sections were obtained by Prof. A.E. Hendrickson from the breeding colony of the Primate Center at Bogor Agricultural University, Indonesia, using protocols approved by the University of Washington (Seattle, WA) Animal Care Committee and in compliance with guidelines of the Association for Research in Vision and Ophthalmology. Fetuses were delivered by aseptic caesarean section, euthanized by intraperitoneal overdose of barbiturate, and then enucleated. Eyes were immediately injected with methyl Carnoy’s fixative (methyl alcohol: acetic acid: chloroform, 6:3:1), then fixed whole by immersion for 1–2 h, as described previously [32]. Sections were paraffin-embedded then cut at 8 μm. Only sections passing through the optic disc and foveal region were used for quantitative analysis in this study.

*Eph-A6* and *PEDF* were cloned from PCR products using total RNA of human fetal retinas, the pGEM^®^-T DNA vector system and JM109 competent cells (#A3600; Promega, Madison, WI). A DIG RNA Labeling Kit SP6/T7 (#1175025; Roche, Basel, Switzerland) was used to transcribe linearized plasmid and generate DIG-labeled antisense and sense probes. The *Eph-A6* and *PEDF* riboprobes were hybridized overnight at 55 °C and 58 °C, respectively, and then washed in saline sodium citrate (pH 7.4) at 65 °C and 58 °C, respectively. Paraffin sections of fetal macaque retina were processed as described previously [16].

## Results

The RNA extracted from human fetal retinas and hybridized to the arrays was of high quality and purity (Table 3). Microarray quality controls were within manufacturer recommendations, and control values were comparable between arrays (Table 3). In addition, on all arrays the average signal values of the poly(A) RNA and hybridization controls increased relative to the order of spike concentrations (*lys*, *phe*, *thr*, *dap* and *bioB*, *bioC*, *bioD*, *cre,* respectively) indicating efficient labeling and hybridization of all samples. The full set of microarray data has been deposited with the NCBI Gene Expression Omnibus repository under accession number GSE12621.

**Table 3:** RNA integrity and microarray quality controls

	**RNA integrity**			**Microarray quality controls**			
Tissue (specimen)	RIN^#^	260/280		Average background	Noise (Raw Q)	GAPDH 3′/5′ ratio	% present calls
						
Macula (1)	8.9	2.03	89.8		3.6	1.09	51.4
Nasal (1)	9	2.03	89.3		3.55	1.15	50.8
Surround (1)	9.3	2	88.4		3.54	1.1	51.5
Macula (2)	9.5	2.04	90.8		3.6	1.09	50.1
Nasal (2)	9.9	1.96	82.8		3.32	1.12	50.9
Surround (2)	9.2	2.08	91.8		3.62	1.14	50.6
Macula (3)	9.1	1.97	80		3.05	1.21	51.9
Nasal (3)	9	1.97	73.4		2.83	1.16	55.5
Surround (3)	8.7	2.02	59.7		2.43	1.05	54.2
Macula (4)	9	1.85	53.9		2.19	1.13	54.6
Nasal (4)	9.3	1.97	57.5		2.37	1.14	54.9
Surround (4)	8.8	2.06	64.9		2.59	1.15	55.7

Integrity measures of RNA samples showed the samples were of appropriate purity for hybridization to microarrays. The RIN was high, ranging from 8.4 to 9.9, and the 260/280 ratios were also high, ranging from 1.85 to 2.08. Microarray quality controls were within manufacturer recommendations and control values were comparable between arrays. The sharp (hash mark) indicates that the RNA integrity number (RIN) ranges from 1 to 10, with 10 indicating no RNA degradation, relative to a database of approximately 1,300 total RNA samples of varying levels of integrity and derived from various tissues from human, rat and mouse [23].

We generated two lists of differentially expressed genes: macula versus surround; and macula versus nasal. We selected a p-value cut-off of <0.01 which gave a false discovery rate (step up method) of 14%. Using DAVID, we found the differentially expressed genes were clustered according to their functional roles, or biological process, generating over 200 gene ontology clusters. Small clusters were consolidated under their parent categories to generate a simplified list (Table 4) that shows the 15 most highly represented biological processes in the microarray data sets. The largest cluster identified includes genes involved with cell metabolism (macula compared to surround: 1,520 genes; macula compared to nasal: 1,059 genes). Differential expression of genes in this cluster, including for example medium- and long-wavelength sensitive cone opsins, glucose phosphate dehydrogenase, and retinol dehydrogenase, is most likely due to the fact that cells in the macula exit the cell cycle and become mature neurons ahead of the rest of the retina. Other categories in which differential gene regulation was expected (because of the more highly differentiated state of macula versus non-macular neurons at 19–20 WG) and confirmed, include neurotransmitter transport (GO:0015813, GO:0006836), secretion (GO:0007269), and signaling (GO:0007215); cell cycle (GO:0007049), cell proliferation (GO:0008283), and division (GO:0051301); and response to light stimulus (GO:0009416).

**Table 4:** Highly represented clusters of genes in the differential expression data

**Gene Ontology ID**	**Biological Process**	**Number of genes**	
		**Macula versus surround**	**Macula versus nasal**
GO:0008152	metabolism	1,520	1,059
GO:0065007	biological regulation	935	883
GO:0010467	gene expression	661	253
GO:0016043	cellular component organization and biogenesis	569	537
GO:0051179	localization	370	532
GO:0007154	cell communication	368	669
n/a^#^	cell proliferation and division	358	355
GO:0032502	development	334	612
GO:0007155	cell adhesion	162	176
GO:0003008	system process	147	170
GO:0050896	response to stimulus	126	136
GO:0046903	secretion	81	64
GO:0040007	growth	64	66
GO:0050953	sensory perception of light stimulus	51	51
GO:0007059	chromosome segregation	30	33

The high representation of genes related to biological processes such as “metabolism” likely reflects the more advanced state of macular development relative to non-macular retina. The sharp (hash mark) indicates that this term is a combination of cell cycle (GO:0007049), cell proliferation (GO:0008283) and cell division (GO:0051301); Abbreviation: Gene Ontology (GO).

In our further analyses we restricted the categories of gene ontology terms to exclude differentially regulated genes associated with the precocious development of the macula. Of the genes reported in Table 4, 52% of the “macula compared to surround” list of differentially expressed probe sets, and 43% of the “macula compared to nasal” list were thus eliminated, leaving a total of 1,788 differentially expressed genes in the “macula versus surround” list and 2,038 in the and “macula versus nasal” list. Further clustering of the restricted lists revealed genes that clustered into five common and highly represented KEGG pathways between the two lists: axon guidance (hsa04360), regulation of actin cytoskeleton (hsa04810), extracellular matrix-receptor interaction (hsa04512), focal adhesion (hsa04510), and ABC transporters-general (hsa02010). The axon guidance KEGG pathway was the most highly represented in the “macula *cf* surround” list. Table 5 lists 25 genes with previously identified roles in axon guidance common to both lists. Seven of the 35 probe sets listed interrogate members of the semaphorin/plexin/neuropilin gene family and six interrogate members of the Eph/ephrin gene family.

**Table 5:** Differentially regulated axon guidance genes, p<0.01

**Gene title**	**Gene symbol**	**DE^#^ (%) Macula versus surround**	**DE^#^ (%) Macula versus nasal**	**Affymetrix Probe Set ID**
actin binding LIM protein 1	*ABLIM1*	33.12	19.67	200965_s_at
Rho guanine nucleotide exchange factor (GEF) 12	*ARHGEF12*	20.26	27.86	201334_s_at
cell division cycle 42 (GTP binding protein, 25 kDa)	*CDC42*	47.89	60.9	214230_at
calcium binding protein P22	*CHP*	34.91	23.52	214665_s_at
chemokine (C-X-C motif) receptor 4	*CXCR4*	−48.13	−49.1	209201_x_at
chemokine (C-X-C motif) receptor 4	*CXCR4*	−51.04	−53.58	211919_s_at
chemokine (C-X-C motif) receptor 4	*CXCR4*	−45.38	−49.6	217028_at
ephrin-A4	*EFNA4*	−32.41	−42.61	205107_s_at
Eph receptor A6	*EPHA6*	14.76	19.94	1561396_at
Eph receptor B1	*EPHB1*	62.04	63.27	210753_s_at
Eph receptor B1	*EPHB1*	53.66	57.47	211898_s_at
Eph receptor B1	*EPHB1*	65.06	69.8	230425_at
Eph receptor B2	*EPHB2*	−34.75	−45.42	210651_s_at
FYN oncogene related to SRC, FGR, YES	*FYN*	17.01	16.65	216033_s_at
guanine nucleotide binding protein (G protein), alpha inhibiting activity polypeptide 3	*GNAI3*	−26.52	−18.49	201179_s_at
integrin, beta 1	*ITGB1*	−32.93	−36.94	1553678_a_at
integrin, beta 1	*ITGB1*	−27.88	−31.33	211945_s_at
NCK adaptor protein 1	*NCK1*	−26.42	−17.45	204725_s_at
neuropilin 1	*NRP1*	−39.47	−49.84	212298_at
netrin G1	*NTNG1*	92.48	272	236088_at
p21 protein (Cdc42/Rac)-activated kinase 3	*PAK3*	38.02	40.06	214607_at
p21 protein (Cdc42/Rac)-activated kinase 7	*PAK7*	40.33	45.9	213990_s_at
plexin C1	*PLXNC1*	−58.01	−60.43	206470_at
plexin C1	*PLXNC1*	−74.36	−67.57	206471_s_at
protein phosphatase 3 (formerly 2B), catalytic subunit, beta isoform	*PPP3CB*	20.31	21.65	202432_at
protein phosphatase 3 (formerly 2B), catalytic subunit, gamma isoform	*PPP3CC*	32.46	48.15	207000_s_at
protein phosphatase 3 (formerly 2B), catalytic subunit, gamma isoform	*PPP3CC*	35.88	42.04	32541_at
semaphorin 3D	*SEMA3D*	66.94	44.71	215643_at
semaphorin 4F	*SEMA4F*	39	46.44	210124_x_at
semaphorin 4F	*SEMA4F*	14.5	12.59	211157_at
semaphorin 4F	*SEMA4F*	44.54	39.42	228660_x_at
slit homolog 2 (Drosophila)	*SLIT2*	52.22	67.76	209897_s_at
slit homolog 2 (Drosophila)	*SLIT2*	57.58	65.8	228850_s_at
SLIT-ROBO Rho GTPase activating protein 2	*SRGAP2*	52	37.55	213329_at
unc-5 homolog D (C. elegans)	*UNC5D*	166.4	191.62	231325_at

Genes associated with the precocious development of the macula were excluded from further analysis. In the adjusted microarray data sets there was a high representation of genes associated with the axon guidance KEGG pathway. Sharp (hash mark) indicates that differential expression (DE) is the ratio of expression levels at the macula compared with nasal/surround, expressed here as a % value. For example, 50% DE indicates a 1.5 fold upregulation, and −50% DE indicates a 1.5 fold down-regulation.

To identify levels of expression of genes involved in the regulation of angiogenesis, we assembled the gene expression data for the 17 negative regulators of angiogenesis identified in the Gene Ontology Consortium, and for isoforms of the major retinal angiogenic factor, *VEGF* (Appendix 1). There was no significant differential regulation of any of the isoforms of *VEGF* detectable by the microarrays (Appendix 1). Of 17 negative regulators of angiogenesis, three—*PEDF*, natriuretic peptide precursor B (*NPPB*), and collagen type IVα2—showed statistically significant fold-changes (p<0.01) in one or both sets of data (Table 6).

**Table 6:** Levels of expression of anti-angiogenic genes in developing human retina, p<0.01

**Gene title**	**Gene symbol**	**Macula versus surround**		**Macula versus nasal**		**Affymetrix Probe Set ID**
		p=	DE**^#^ (%)**	p=	DE**^#^ (%)**	
pigment epithelium derived factor (serpin peptidase inhibitor, clade F member 1)	*PEDF*	0.003	49.52	0.004	46.62	202283_at
natriuretic peptide precursor B	*NPPB*	0.003	28.57	0.002	33.05	206801_at
collagen, type IV, alpha 2	*COL4A2*	0.151	−21.16*	0.005	−59.72	211964_at
collagen, type IV, alpha 2	*COL4A2*	0.033	−35.56*	0.001	−85.44	211966_at

*PEDF, NPPB*, and *COL4A2* were the only anti-angiogenic genes, out of 17 known negative angiogenic regulators, to show significant differential expression between the macula and surrounding retina. Non-significant fold changes (*p>0.01) are indicated.

**Figure 2:**
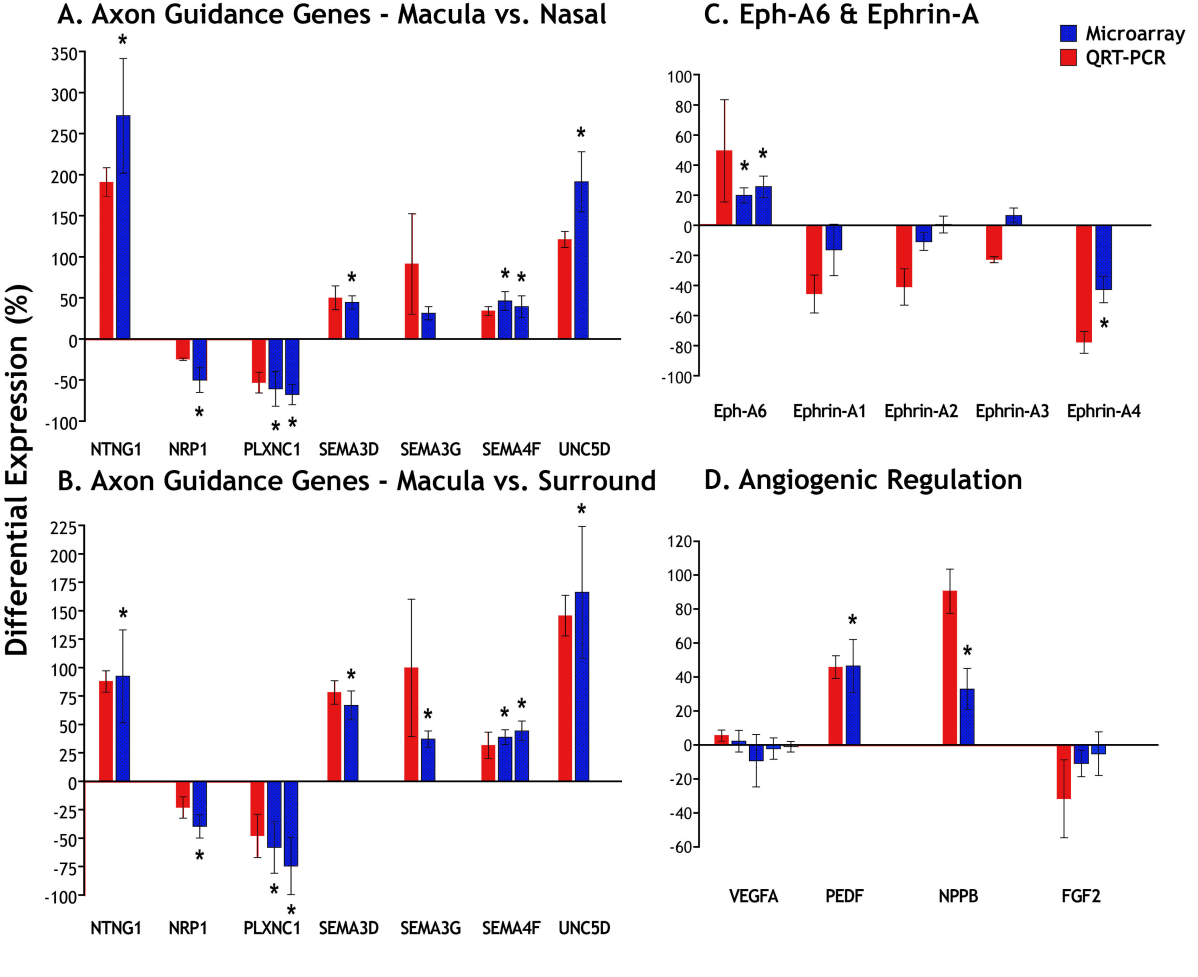
Measures of differential gene expression. Microarray data are indicated by blue bars and QRT–PCR data by red bars. We selected differentially regulated genes (*p<0.01) identified by the microarray, binding partners of those differentially regulated genes, as well as five other relevant genes of interest: *SEMA3G, FGF2, VEGF,* and *ephrin-A2* and *-A3*. **A**, **B:** Selected axonal guidance genes. These genes were significantly upregulated or downregulated in the macula compared to either the nasal (**A**) or surround (**B**) samples, with the exception of *SEMA3G*, which was significantly upregulated only in the macula versus surround comparison (**B**). **C:** QRT–PCR indicates strong differential regulation of *Eph-A6* and its ligands *ephrin-A1 and -A4* in the macula compared with nasal samples. The QRT–PCR results showed stronger trends in differential expression compared to the microarray, with the exception of *ephrin-A3*, which QRT–PCR suggests is not differentially expressed. Similar data were obtained from macula versus surround comparisons. **D:** The QRT–PCR confirmed significant upregulation of *PEDF* and *NPPB* in the macula compared with the nasal sample. Both the microarray and QRT–PCR findings indicate no differential regulation of *VEGFA*, the major isoform expressed in the retina. The small downregulation of *FGF2* in the microarray and QRT–PCR data are consistent with our previous findings that show a downregulation of *FGF2* in macular cones, but not in other layers of the retina, by in situ hybridization [32]. Note that the standard error of the microarray fold changes is derived from the distribution of fold changes measured across the four specimens. Abbreviations: fibroblast growth factor 2 (*FGF2*); natriuretic peptide precursor B (*NPPB*); neuropilin 1 (*NRP1*); netrin G1 (*NTNG1)*; pigment epithelium derived factor (*PEDF*); plexin C1 (*PLXNC1*); semaphorin 3D (*SEMA3D*); semaphorin 3G (*SEMA3G*); semaphorin 4F (*SEMA4F*); unc-5 homolog D (*C. elegans*; *UNC5D*); vascular endothelial growth factor A (*VEGFA*).

To test the veracity of the microarray data, we evaluated the expression of eleven genes from Table 5 and Table 6 and five additional genes of interest (including binding partners of significantly regulated genes, closely related genes, and significant angiogenic factors) using cDNA from retinas aged between 17 and 20 WG by QRT–PCR. Figure 2 shows the mean relative level of expression±standard error determined by QRT–PCR (red columns) compared with differential expression data from the microarray data (blue columns). The results show strong consensus between the microarray and QRT–PCR data for virtually all genes. The graphs confirm downregulation of neuropilin 1 (*NRP1*) and plexin C1 (*PLXNC1*) in the macula, and upregulation of the other selected genes in the macula. Comparison of fold-changes in “macula compared to surround” and “macula compared to nasal” (Figure 2A,B) revealed no significant difference between the two data sets. In subsequent QRT–PCR experiments we compared expression of *ephrin A1-A5* and *Eph-A6* (Figure 2C), and four regulators of angiogenesis (Figure 2D) in cDNA from macula and nasal locations. The results indicated strong upregulation of *Eph-A6* in the macula, and downregulation of its 2 ligands, *ephrin-A1* and -*A4*, as well as *ephrin-A2* and -*A3*. *Ephrin-A5* did not amplify reliably (data not shown). We found no differential regulation of *VEGFA* by QRT–PCR, consistent with the microarray data. We detected evidence of borderline downregulation of fibroblast growth factor 2 (*FGF2*) in the macula, consistent with a prior report [32]. Significantly, QRT–PCR confirmed strong upregulation of two negative regulators of angiogenesis: *NPPB* [33] and *PEDF* [34] (Figure 2D).

**Figure 3:**
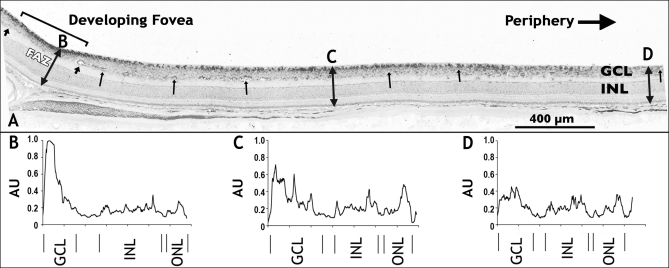
In situ hybridization for *Eph-A6* mRNA in fetal macaque retina. Small arrows indicate the profiles of retinal vessels, and the thicker arrows (to the left) mark the vessels on the margin of the foveal avascular zone (FAZ). Double-headed arrows show optical densitometry transects (**B-D**). **A:** *Eph-A6* mRNA expression is shown in a section through the developing fovea in a retina from a macaque at Fd 115. There are high levels of *Eph-A6* expression (dark reaction product) in the inner part of the ganglion cell layer (GCL) at the developing fovea and lower levels of *Eph-A6* expression in the GCL at progressively more peripheral locations, consistent with both the QRT–PCR and microarray findings. A second gradient of *Eph-A6* mRNA expression across the depth of the GCL at foveal and parafoveal locations (left and central of **A**) was also detected, as shown by optical densitometry profiles at **B, C,** and **D**. **B:** A transect through the retina within the FAZ shows peak expression of *Eph-A6* mRNA in the inner GCL, declining to low levels in the outer GCL. **C:** A weaker gradient was detected across the GCL about 1.5 mm from the incipient fovea, with peak levels approximately 70% of the peak value in the fovea. **D:** *Eph-A6* expression was generally lower in the periphery, although slightly higher levels are present in the GCL compared with the other layers. Values in **C** and **D** are normalized to the peak intensity in **B**. Abbreviations: arbitrary units (AU); inner nuclear layer (INL); outer nuclear layer (ONL).

**Figure 4:**
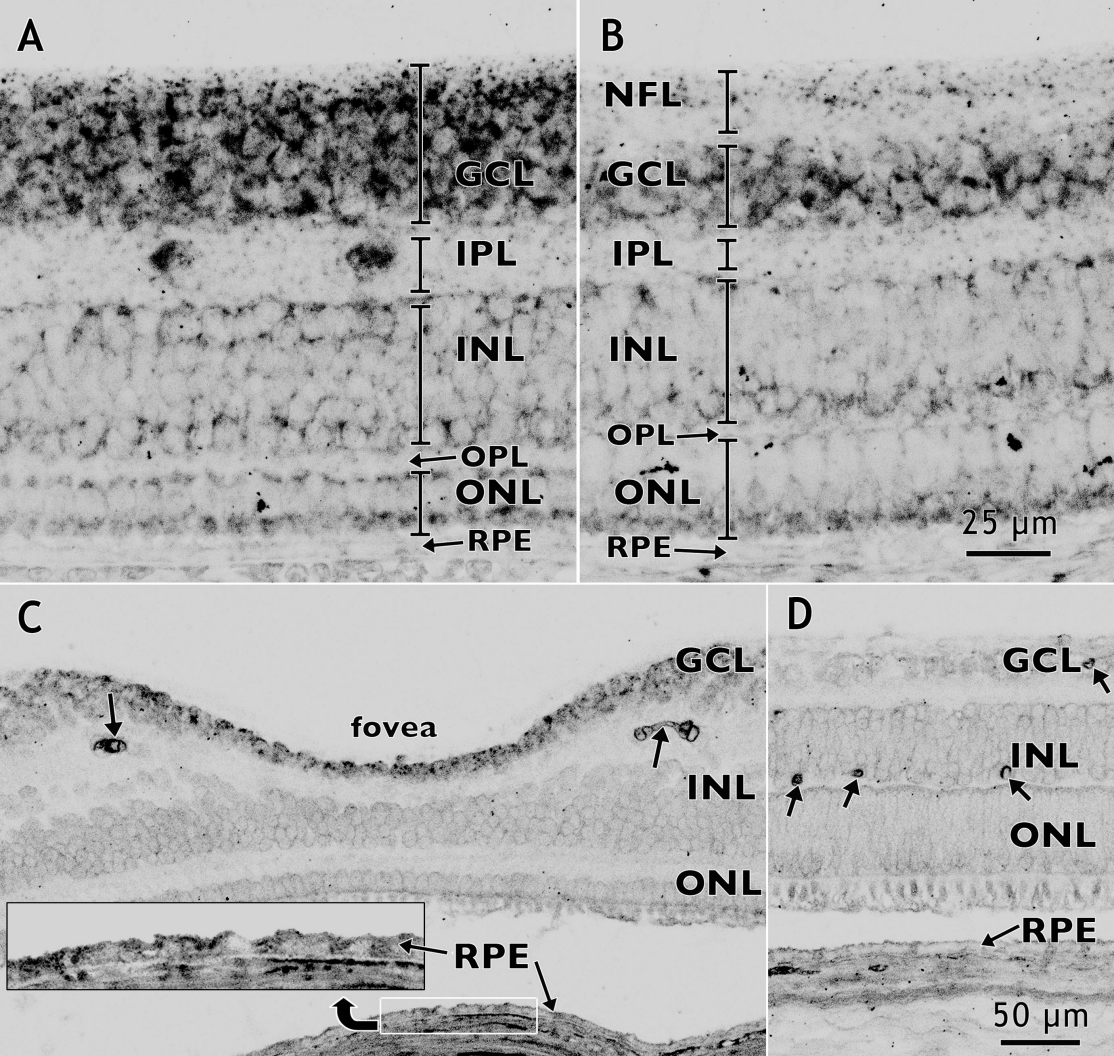
In situ hybridization for *PEDF* mRNA (dark reaction product) in fetal macaque retina. Layers of the retina are indicated by the vertical brackets. Vessel profiles are indicated in **C** and **D** by oblique arrows. **A**, **B:** At Fd 80, higher levels of *PEDF* expression were detected in the GCL at the incipient fovea (**A**) compared with a location near the optic disc (**B**). *PEDF* levels were low in the RPE at Fd 80. Labeled cells in the IPL of **A** are most likely displaced ganglion cells which are common in central retina at this age. **C**, **D:** In the Fd 150 fovea (**C**), relatively high levels of *PEDF* mRNA are present in the GCL compared with levels in the GCL near the optic disc (**D**). Inset shows increased *PEDF* expression in the RPE at Fd 150 compared with Fd 80 (see **A** and **B**). Abbreviations: ganglion cell layer (GCL); inner nuclear layer (INL); inner plexiform layer (IPL); outer nuclear layer (ONL); outer plexiform layer (OPL); nerve fiber layer (NFL); retinal pigmented epithelium (RPE).

We selected one axon guidance gene, *Eph-A6*, and one negative regulator of angiogenesis, *PEDF*, to examine their retinal distributions by in situ hybridization. Figure 3A shows a macaque retina at Fd 115, equivalent to around 25 WG in the human, hybridized to show the retinal distribution of *Eph-A6* mRNA. In this retina, and others from macaques aged Fd 110 to postnatal 3 months (data not shown), *Eph-A6* mRNA expression was high in the macula, reaching a peak at the developing fovea, and was lower in the periphery [35]. We also detected a graded expression of *Eph-A6* across the thickness of the ganglion cell layer (GCL) that is high in the inner GCL and low in the outer GCL at foveal and parafoveal locations, but not peripheral locations (Figure 3). In situ hybridization for *PEDF* in developing macaque retinas (Figure 4A-D) shows *PEDF* mRNA in the GCL and RPE, consistent with previous reports [36], although we found low to very low *PEDF* expression in the developing photoreceptors of fetal macaque retinas (Figure 4A-D). *PEDF* mRNA expression was low in the RPE at Fd 80 (about 17 WG in humans), but higher at Fd 150 (about 32 WG in humans; Figure 4A,B compared to Figure 4C,D). We detected *PEDF* expression at higher levels in the GCL of central retina, compared with non-central retina, at both Fd 80 and Fd 150. This gradient of expression of *PEDF* in the GCL is consistent with the microarray and QRT–PCR data.

## Discussion

To our knowledge, this is the first investigation into the differential gene expression in the developing macula. Previous differential expression studies have used adult tissue [37] or have not specifically investigated the developing macula [38].

### Differential expression of anti-angiogenic factors

This is the first report of upregulation of anti-angiogenic factors in the primate macula: the widely recognized inhibitor *PEDF*, and a novel anti-angiogenic regulator for the retina, *NPPB*. The apparent downregulation of collagen type IVα2 in the macula is most likely due to the presence of collagen-invested retinal vessels in the non-macular samples. Our findings are consistent with a role for both *PEDF* and *NPPB* (also known as brain natriuretic peptide) in the definition of the avascular area in the central macula, which, at a later stage of development, will include the fovea centralis.

*PEDF* is a neurotrophin [39] and has potent anti-angiogenic effects in human [36] and other mammalian retinas [40,41]. The anti-angiogenic effects of *PEDF* occur through stimulation of the Fas/Fas ligand-mediated apoptotic pathway which targets the endothelial cells of immature vessels, and may also downregulate the caspase-8 inhibitor, c-FLIP [42]. Both of these effects reduce the survival of new endothelial cells, even in the presence of pro-angiogenic factors. *VEGF*-induced angiogenesis can be directly inhibited by *PEDF* via the intracellular proteolysis of *VEGFR-1* or inhibition of its phosphorylation [43].

Less is known about *NPPB* in the retina. *NPPB* is a cardiac hormone and influences several systemic functions such as body fluid homeostasis, blood pressure, cardiac function, bone growth, and vascular tone [44]. *NPPB* has been amplified from adult human retina [33] and the protein localized to ganglion, Müller [33,45], and amacrine cells [46] in humans and rats. Natriuretic peptides can affect *VEGF*-induced angiogenesis by inhibiting certain signaling molecules such as ERK, JNK, and p38 members of the MAP kinase family through stimulation of a cyclic GMP signaling cascade (natriuretic peptide receptors NPR-A and -B) or through a less well understood mechanism involving NPR-C [44,47-49].

Type IV collagen is an important structural component of basement membrane and is required for blood vessel formation [50]. The 24 kDa C-terminal domain of α2 chains of type IV collagen, also known as canstatin, inhibits endothelial cell proliferation and migration, and induces other anti-angiogenic mechanisms including Fas ligand expression (reviewed in [51]). One would expect type IV collagen to be restricted to the more mature retinal vessels, which at 19–20 WG, are mainly in nasal and temporal superior/inferior retina; very few vessels would have been biopsied in our macular samples.

### Differential expression of guidance factors

We have also identified 25 axon guidance genes differentially expressed in the macula, including 12 members of the ephrin, semaphorin, slit and netrin families, which are also regulators of vascular development [52]. Significantly, we detect this differential regulation of guidance genes at the macula 8–12 weeks after macular ganglion cells differentiate and send out axons, and 3–4 weeks after the majority of retinal axons have exited the retina [30]. Retinal neurons are generated in a stereotyped sequence [53] and along a centro-peripheral gradient [30,54], such that ganglion cells and cones of the incipient fovea are among the first neurons generated in the primate retina. Ganglion cell axons are observed in the optic stalk from around 8 WG in humans [55] (reviewed in [56]), reach a peak number at 16–17 WG, and stabilize at adult values between 25 and 30 WG, approximately [57]. Although for the most part, axon guidance within the retina and visual targets has not been studied in primate retina (see, however, Lambot et al., [58]), we know from studies of other species that a range of axon guidance factors are expressed in the retina during the period of axon outgrowth, to guide axons to the optic disc [59,60], and regulate targeting of their axon terminals by interaction with their binding partners in the primary visual nuclei [61-65]. Because axons of macular ganglion cells are among the first to enter the optic nerve—estimated between 8 and 14 WG—we interpret the sustained expression of guidance factors in the macula at 19–20 WG, as detected in the present study, to be primarily related to vascular patterning, rather than axon guidance.

Many genes originally identified by their involvement in axon patterning are also implicated in vascular patterning. The semaphorin-plexin family of genes share with *VEGFA* the capacity to bind neuropilin 1, expressed by both blood vessels and axons. Class 3 semaphorins are also known to have a repellent effect during vascular morphogenesis via interactions with integrins [66]. Eph receptors and their ephrin ligands have key roles in axon guidance, provide guidance cues for endothelial cells during development, are involved in assembly and maintenance of vascular networks, and arteriovenous differentiation [67]. Netrin is a potent vascular mitogen [22] and has a role in repelling developing vessels via interactions with the *UNC5* receptor [68], while *Slit2* is implicated in endothelial cell migration [52]. Of particular interest are the repellent effects of netrin-*UNC5* interactions [68] and Eph-ephrin signaling [69] on developing vessels as well as axons during development. We propose that a graded expression of genes involved in repellent signaling that is centered on the fovea during development - similar to the one we report here for *Eph-A6* (Figure 3) - retards the growth of vessels into the central region of the retina, and contributes to definition of the foveal avascular area (in preparation).

### Significance

The spatial resolving power of the fovea is directly attributable to cone inner segment spacing which reaches a peak of 100,000 to 300,000 mm^−2^ [70] at the geometric center of the avascular area, and corresponds with the central fovea (foveola) in adult retinas. Studies using adaptive optics show that while most individuals do not use the area of peak cone density for fixation, the mean displacement of the fixation point from the peak cone density (50 µm) [71] places it comfortably within the average-sized avascular area (500–700 µm). Shadowing of the photoreceptor mosaic causes angioscotomas in the visual cortex [72]. Thus, reduction in the diameter of macular vessels, and an absence of vessels overlying the region of peak cone density at the fovea, might be regarded as an adaptive advantage that favors optimal spatial acuity. It is possible that the mechanisms that mediate specialization of the macular vessels, and which define the foveal avascular area, “piggyback” on axon guidance mechanisms in effect earlier in development. One hypothesis is that the guidance factors that repel ganglion cell axons away from central retina into the arcuate axon bundles and optic nerve head also guide vessels out of the optic nerve head and repel them from the foveal region, at a slightly later phase of development.

A series of findings suggest that definition of an avascular area within the macula is required for subsequent formation of a fovea. First, developmental studies indicate that in macaques and marmosets the foveal avascular area is defined before the fovea begins to form [18,20]. Second, optical coherence tomography in humans indicates that absence of an avascular area is associated with foveal hypoplasia, or absence of a foveal depression [14,15]. Third, finite element analysis predicts that differential elasticity in the foveal region, resulting from a local absence of retinal vessels, is sufficient to promote development of the foveal depression due to growth-induced stretch [73]. Our present results suggest that both anti-angiogenic and axon guidance factors have key roles in regulating vascular growth in the macula and in specifying the foveal region as a vascular “no go” area, and are thus indirectly implicated in the later formation of the fovea centralis.

Expression of anti-angiogenic factors in the macula during development, and guidance factors that have roles in regulating vascular growth in the macula through repellent mechanisms, suggests there may be a biological conflict of interest in the primate macula; between high density neural networks, with correspondingly high levels of energy demands, on the one hand, and adaptive pressure to constrain blood vessel growth to avoid shadowing of photoreceptors, on the other. These findings may be useful to understand the vulnerability of the macula to degeneration and to develop new therapeutic strategies to inhibit neovascularization.

## Appendix 1. Levels of expression of angiogenic and anti-angiogenic genes in developing human retina

Differential expression data of the 17 negative regulators of angiogenesis identified in the Gene Ontology Consortium, and the isoforms of *VEGF*. To access the data, click or select the words “Appendix 1.” This will initiate the download of a compressed (pdf) archive that contains the file.
